# Host–Guest Complexes

**DOI:** 10.3390/ijms232415730

**Published:** 2022-12-12

**Authors:** Juan C. Mejuto, Jesus Simal-Gandara

**Affiliations:** 1 Department of Physical Chemistry, Faculty of Science, University of Vigo, Ourense Campus, E32004 Ourense, Spain; 2 Nutrition and Bromatology Group, Department of Analytical and Food Chemistry, Faculty of Science, University of Vigo, Ourense Campus, E32004 Ourense, Spain

Host–guest complexes, also known as inclusion complexes, are supramolecular structures [1,2] composed of two or more molecules or ions that are maintained through noncovalent interactions in a reversible way (Figure 1).

The hosts have a cavity that allows them to behave as main host receptors with high affinity and selectivity for guest molecules. Macrocycles are generally used as host molecules (Figure 2). 

These molecules include cryptands [3], crown ethers [4,5,6], cyclophanes [7], cyclopeptides [8,9], cyclodextrins [10,11,12], resorcin-arenes [13], cucurbit[n]urils [14,15], calix[n]arenes [16,17,18,19], and pillar[n]arenes [20,21].

In the literature, there are multiple examples of the applicability of these supramolecular systems in numerous fields of science and technology, such as functional materials, catalysts, electronic devices, sensors, and in the pharmaceutical or food industries [22,23,24,25,26].

In this Special Issue, entitled “Host–Guest Complexes”, structural aspects of the formation and stability of these complexes, as well as their characterization and aspects related to their technological applications, are addressed. 

Galmés et al. [27] addressed non-covalent interactions associated with supramolecular compounds from the perspective of theoretical chemistry. In addition to this contribution, associated with the structural and energetic characteristics of host–guest complexes, multiple applications have been studied from a technological point of view, such as drug carriers and drug deliverers [28,29,30], gene delivery for immune-modulating therapy (Bai et al. [28]), inclusion complexes for cancer cells (Raffaini et al. [29]), and crystals as a solid solution for enantiomers (Czapik et al. [30]).

Likewise, corresponding results have been presented with the modification and/or improvement of physicochemical characteristics of substances associated with the use of inclusion complexes. This takes advantage of synergistic effects [31,32] for improving targeting and local anesthesia in inflamed tissues (Couto et al. [31]) and both antimicrobial and modulatory activity (de Alemeida-Magalhäes et al. [32]).

In this Special Issue, findings in the field of biotechnology are also presented by García-Pérez et al. [33], such as the presence of host–guest complexes showing an improvement in the production of bioactive compounds [33].

In short, the set of contributions that make up this Special Issue show that host–guest complexes constitute an interesting family of chemical structures whose study is an integral part of innovative current scientific research in the field of supramolecular chemistry. This is due to both the scientific interest they arouse and the multiple and varied applications in the field of new technologies in a large number of industrial sectors.

## Figures and Tables

**Figure 1:**
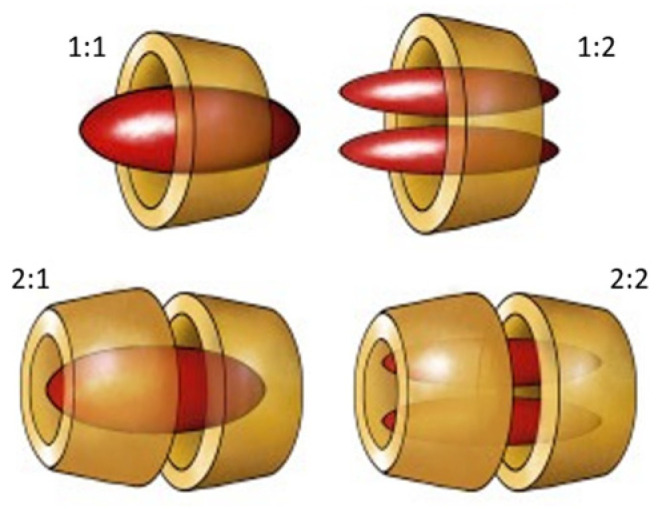
Schematic structure of a host–guest complex with different stoichiometries.

**Figure 2:**
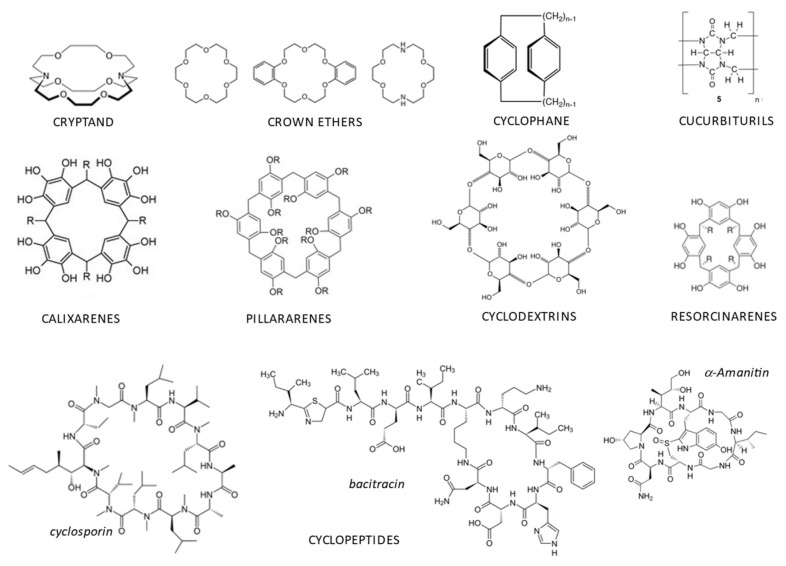
Typical host macrocyclic molecules.
